# Metronidazole-induced encephalopathy in a patient with pyogenic spondylitis: a case report

**DOI:** 10.1186/s12891-018-2255-8

**Published:** 2018-09-18

**Authors:** Kazutaka Mizuta, Motoki Sonohata, Osamu Nozaki, Tomoki Kobatake, Daisuke Nakayama, Tadatsugu Morimoto, Masaaki Mawatari

**Affiliations:** 1 Department of Orthopaedic Surgery, Yanagawa Hospital, 29 Chikushi-machi, Yanagawa, 832-0077 Japan; 2 Department of Orthopaedic Surgery, Faculty of Medicine, Saga University, 5-1-1 Nabeshima, Saga, 849-8501 Japan

**Keywords:** Metronidazole-induced encephalopathy, Antimicrobial, Complication, Magnetic resonance imaging

## Abstract

**Background:**

Metronidazole is an antimicrobial agent commonly used in the treatment of several protozoal and anaerobic infections. Neurotoxicity associated with metronidazole has been rarely reported, and the incidence of metronidazole-induced encephalopathy is unknown. Therefore, the accurate diagnosis of metronidazole-induced encephalopathy is often difficult because of the rarity of the disease.

**Case presentation:**

An 86-year-old woman suffered from pyogenic spondylitis of the lumbar spine. *Parvimonas micra,* a gram-positive anaerobic bacterial species and a resident of the flora of the oral cavity, was identified in the biopsy specimens. Oral administration of metronidazole (1500 mg/day) was initiated. Forty-four days after initiating metronidazole (total intake of 66 g), she complained of tingling sensations in the upper limbs. After 4 days, she complained of additional symptoms including sensory disturbance of the tongue, dysarthria, and deglutition disorder. Characteristic brain magnetic resonance imaging findings on T2-weighted fluid-attenuated inversion recovery and diffusion-weighted imaging led to the diagnosis of metronidazole-induced encephalopathy. Metronidazole was discontinued, and her neurological symptoms improved 10 days after discontinuation. At 14 days after discontinuation of oral metronidazole, abnormal findings on diffusion-weighted imaging almost disappeared.

**Conclusions:**

With the possibility of needing to prescribe metronidazole in the orthopedic field for the treatment of various infections, orthopedic surgeons are likely to encounter cases of metronidazole-induced encephalopathy. Thus, they should be able to recognize the condition and its potential complications. With increased awareness, early diagnosis with magnetic resonance imaging and discontinuation of metronidazole may become feasible when such patients are referred. Our report presents a detailed account of such a case, which may help in the early diagnosis and treatment of patients with metronidazole-induced encephalopathy. Furthermore, we recommend that patients treated with metronidazole should undergo careful and constant surveillance after starting antibiotic therapy.

## Background

Metronidazole is an antimicrobial agent commonly used to treat several protozoal and anaerobic infections. Especially, metronidazole is widely used as a standard therapeutic agent for *Clostridium difficile*-associated infection [1]. It has been prescribed for conditions, such as Crohn’s disease, intra-abdominal abscess, *Helicobacter pylori* infection, hepatic encephalopathy, and recurrent pyogenic cholangitis [2]. Although it is well tolerated in common settings, patients may experience serious neurological side effects with both long- and short-term use. Its side effects include an ataxic gait, peripheral neuropathy, cerebellar dysfunction, dysarthria, vestibulotoxicity, cochleotoxicity, seizures, encephalopathy, and visual impairment [3–9]. Neurotoxicity with metronidazole has been rarely reported, and the incidence of metronidazole-induced encephalopathy (MIE) is unknown. Typical MIE is diagnosed on the basis of abnormal signals on magnetic resonance imaging (MRI) involving the cerebellar dentate nuclei. In addition, many structures, including the corpus callosum, inferior colliculus, midbrain, pons, medulla, and bilateral subcortical white matter, are involved; however, the basal ganglia are less involved [10–15]. In addition, making the appropriate diagnosis is often difficult because of the rarity of the disease in the orthopedic field. Here, we report a rare case of MIE in a patient with pyogenic spondylitis.

## Case presentation

**Fig. 1:**
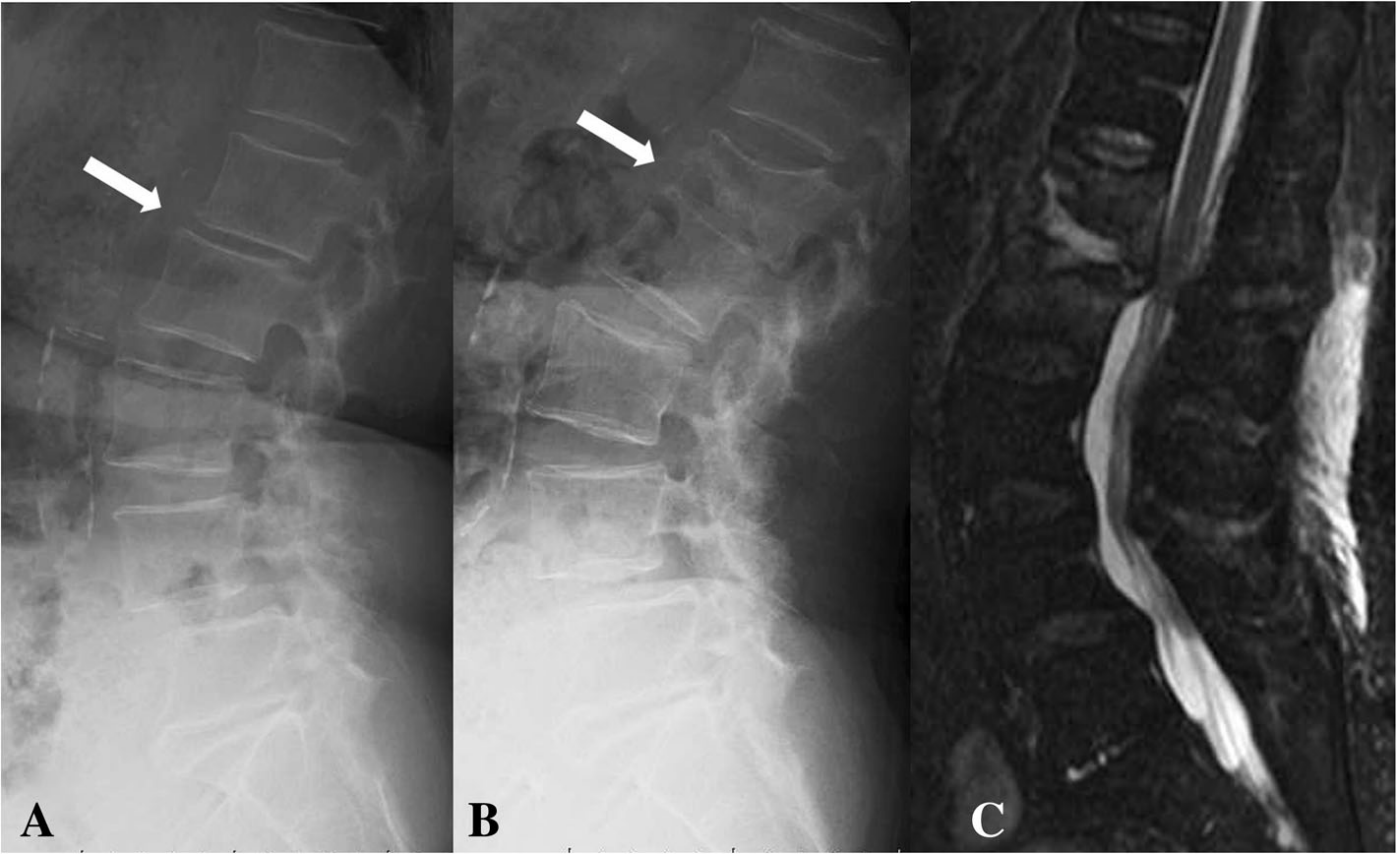
**a** Roentgenogram of the lateral lumbar vertebra at the first visit. A slightly narrow L1/2 is observed (white arrows). **b** Roentgenogram of the lateral lumbar vertebra at 1 month after the first visit. Collapse of L1 and absorbable changes at the caudal side of L1 are observed (white arrows). **c** T2-weighted fat-suppression magnetic resonance image. Effusion and edema are observed in L1 and L2

**Fig. 2:**
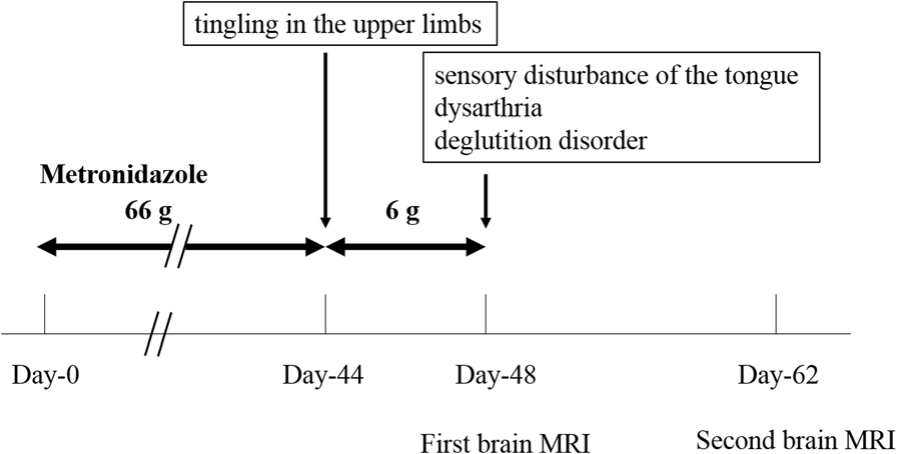
Time line after the initiation of metronidazole

**Fig. 3:**
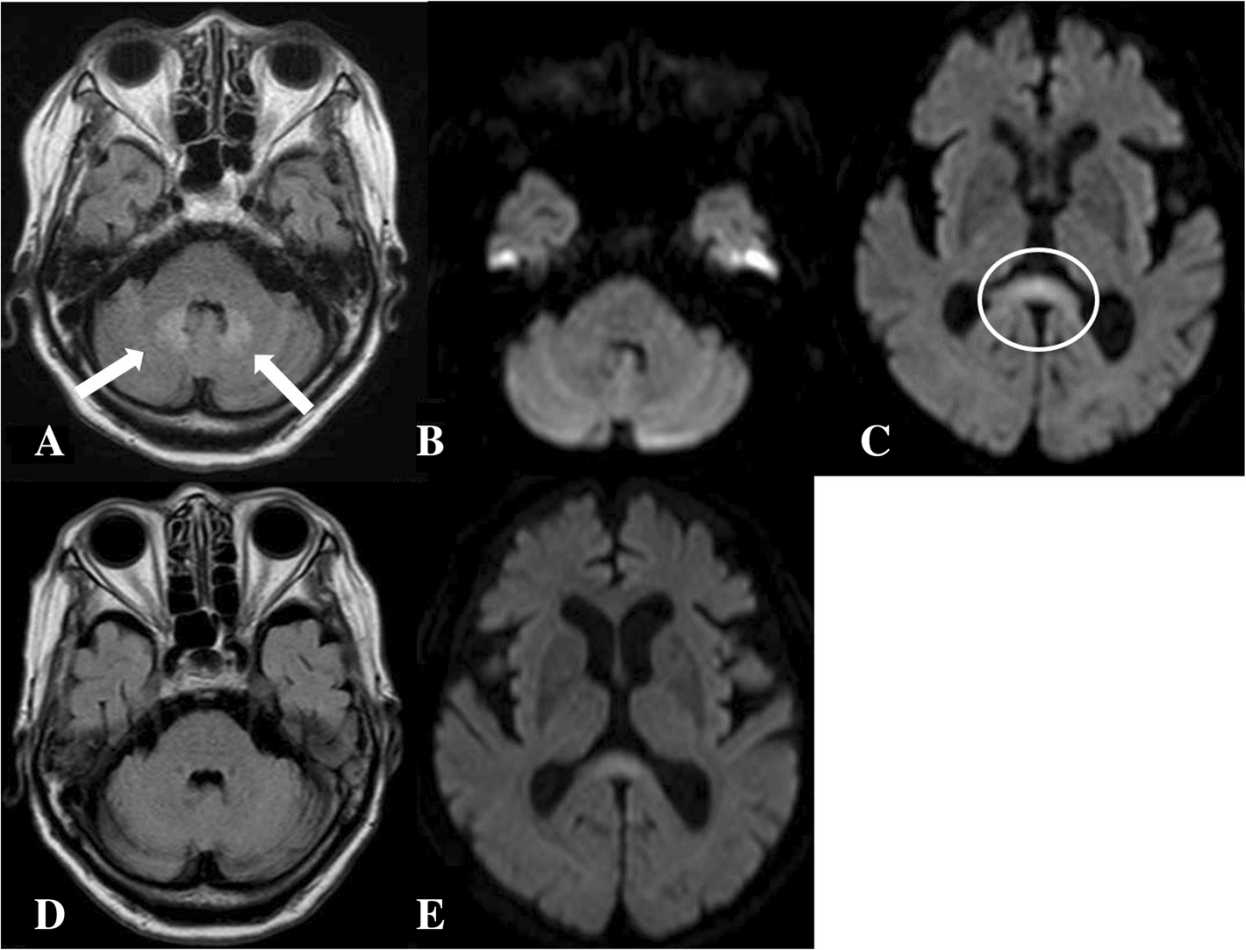
**a** Brain magnetic resonance imaging. T2-weighted fluid-attenuated inversion recovery (FLAIR) imaging performed at 4 days after symptom onset shows hyperintense areas in the bilateral basal dentate nuclei (white arrows). **b** Diffusion-weighted imaging (DWI) performed 4 days after symptom onset showed an almost normal intensity in the bilateral basal dentate nuclei. **c** DWI performed 4 days after symptom onset revealed a hyperintense T2 signal with compatible cytotoxic edema (white circle). **d** At 14 days after discontinuation of oral metronidazole, abnormal findings on T2-weighted FLAIR imaging completely disappeared. **e** At 14 days after discontinuation of metronidazole, the abnormal findings on DWI almost disappeared

An 86-year-old woman with back pain was referred to our hospital. At the time of presentation, she had no fever and no pain at rest. Roentgenography findings were relatively normal with slight narrowing at L1/2 (Fig. 1a). She had no known drug allergies. She was administered analgesics and was kept under observation. One month after her first visit to our hospital, her back pain worsened, and her temperature was 37.2 °C. No neurological abnormalities were noted in her lower limbs. Laboratory findings at that time were as follows: C-reactive protein, 11.5 mg/dL (reference, < 0.2 mg/dL); white blood cell count, 7970/mm^3^ (neutrophils, 87.7%; reference, 4000–8000/mm^3^); aspartate aminotransferase, 15 IU/L (reference, 13–33 IU/L); alanine aminotransferase, 13 IU/L (reference, 6–30 IU/L); alkaline phosphatase, 291 IU/L (reference, 115–359 IU/L); and creatinine, 1.5 mg/dL (0.4–0.7 mg/dL). Radiographs of the lumbar spine showed collapse of L1 and an absorbable change in the caudal side of L1 (Fig. 1b). Lumbar MRI confirmed the presence of fluid at the L1/2 disc, edema and destruction of the spinal body of L1/2, and lumbar canal stenosis at the level of L1/2 on fat-saturated T2-weighted images (Fig. 1c). She was diagnosed with pyogenic spondylitis of the lumbar spine and underwent biopsy of the L1/2 disc. *Parvimonas micra,* which is a gram-positive anaerobic bacterial species and a resident of the flora of the oral cavity, was identified in the biopsy specimens. Given that the patient was able to tolerate oral medications and the transfer rate of metronidazole to the blood was similar between oral and intravenous administrations, we decided to administer the medications orally. Oral administration of metronidazole (1500 mg/day) was initiated (Fig. 2). Forty-four days after starting metronidazole (total intake of 66 g), she complained of tingling in the upper limbs. Cervical spinal disorder was suspected, and cervical spinal MRI demonstrated spinal canal stenosis at C3/4, 5/6, and 6/7. After 4 days, she complained of further symptoms, including sensory disturbance of the tongue, dysarthria, and deglutition disorder. Central nervous system disorder was suspected, and brain MRI was performed. Characteristic brain MRI findings on T2-weighted fluid-attenuated inversion recovery (FLAIR) and diffusion weighted imaging (DWI) led to the diagnosis of MIE (Fig. 3a, b). Metronidazole was discontinued, and her neurological symptoms improved 10 days after discontinuation. On MRI performed 14 days after oral metronidazole discontinuation, most of the abnormal findings of MIE disappeared (Fig. 3c, d). Fortunately, follow-up blood tests revealed the absence of inflammatory reactions 5 days before the discontinuation of metronidazole; no antibiotics were administered after discontinuing metronidazole. At 9 weeks after discontinuation of metronidazole, there was no recurrence of pyogenic spondylitis according to the clinical findings or blood sample results.

## Discussion and conclusions

In the orthopedic field, *Staphylococcus aureus* is one of the primary bacteria causing bone and joint infections or periprosthetic joint infections [16]. However, reports on infections caused by oral bacteria in the orthopedic field have increased in recent years [17, 18]. Many kinds of anaerobic bacteria exist in the oral cavity, and Walter et al. [16] described that the diagnosis of anaerobic bone and joint infections was underestimated before molecular identification was established. In cases of anaerobic bone and joint infections, antibiotic treatments included amoxicillin, amoxicillin-clavulanic acid, metronidazole, and clindamycin [19]. Neurotoxicity associated with metronidazole presents with a wide spectrum of symptoms, including mental status changes, peripheral neuropathy, weakness, vertigo, nausea, vomiting, and sensory loss [9, 20]. Osteoarthritis infections caused by *Parvimonas micra* are rarely encountered by orthopedic surgeons [21]. Therefore, in the current case, sensitivity tests on antibiotics were not performed adequately; thus, adverse effects of metronidazole administration were not adequately recognized. Our literature search yielded no other case reports of MIE occurring during treatment of pyogenic spondylitis. When neurologic symptoms deteriorate during treatment of pyogenic spondylitis with metronidazole, attention should be paid to neurotoxicity, including the possibilities of MIE and peripheral neuropathy caused by metronidazole, in addition to aggravation of pyogenic spondylitis.

However, peripheral neuropathy may appear as a primary symptom, and the diagnosis may be delayed. In the current case, MIE presented as a peripheral neuropathy with a tingling sensation, and the diagnosis of MIE was delayed by 4 days. The proposed mechanism of MIE is thought to involve metronidazole-induced neurotoxicity associated with the oxidation of catecholamine neurotransmitters to semiquinone and nitro anion radicals, which reduce tissue oxygenation and subsequently generate superoxide radicals and hydrogen peroxide. However, the mechanism remains unclear [22].

Metronidazole doses of 45–120 g and treatment durations of 1–12 weeks have been reported to result in MIE [23–25]; however, the doses and durations widely vary. The half-life of metronidazole is 6–8 h; however, the half-life in patients with liver dysfunction or renal dysfunction is approximately 3-folds higher. Accordingly, it is necessary to limit the total cumulative dose to less than 20 g [26]. Our patient had mild renal dysfunction, and the total cumulative dose was 66 g. Therefore, when metronidazole is used, the patient’s neurological symptoms should be monitored closely [26].

The clinical disease concept of MIE was reported over 20 years ago [3–8]; however, MIE-specific MRI findings have been reported recently [10–15]. The brain MRI findings of MIE were categorized according to the apparent diffusion coefficient, which are as follows: minimal (grade 1) indicating symmetric involvement of one lobe (frontal, temporal, parietal, or occipital) without involvement of the corpus callosum, basal ganglia, thalami, or internal capsules; mild (grade 2) indicating symmetric involvement of two lobes, or of one lobe plus symmetric involvement of one of the corpus callosum, basal ganglia, thalami, or internal capsules; moderate (grade 3) indicating symmetric involvement of two lobes plus symmetric involvement of one of the corpus callosum, basal ganglia, thalami, or internal capsules; and severe (grade 4) indicating symmetric, extensive, and confluent involvement of three or all lobes from the ventricular margin to the subcortical white matter, or of two lobes plus symmetric involvement of two of the following: corpus callosum, basal ganglia, thalami, or internal capsules [14, 15].

A direct correlation was found between the high cumulative dose and the lesion severity in the corpus callosum. Moreover, lesions with restricted diffusion and those without restricted diffusion may sometimes have different pathophysiological mechanisms [12]. Previous studies also reported that lesions without restricted diffusion, including those in the cerebellar dentate nucleus, disappeared after metronidazole discontinuation [27, 28]. However, the abovementioned classical finding may or may not be present during the initial stage of the disease [26, 29]. In most cases, MIE is a reversible disease that improves within a few weeks after discontinuation of metronidazole [23, 30]. However, MIE is not always reversible and may result in a fatal outcome [14, 31]. Therefore, correct and early diagnosis is very important in the management of patients with MIE. Thus, when metronidazole is administered, the dose and duration of treatment should be limited.

In the current case, pyogenic spondylitis was treated with metronidazole for 48 days, and no recurrence was observed for 9 weeks. Although the treatment period exceeded six weeks, which is recommended for treating pyogenic spondylitis [32], the follow-up period was as short as 9 weeks.

The incidence of MIE is not very high, and the majority of patients with MIE recover uneventfully. However, occasionally, the symptoms of peripheral neuropathy persist or become severe. In the current case, there were no sequelae, but the patient was not initially accurately diagnosed. MIE has been well described, but it has been rarely reported in the orthopedic literature. In the past, there are only four cases of possible spondylitis due to *Parvimonas micra* [21]; however, there may be more potential cases. Orthopedic surgeons should be responsible in improving their knowledge of the appropriate antibiotics for treating infections caused by *Parvimonas micra* as well as their therapeutic and adverse effects. As orthopedic surgeons are likely to encounter cases of MIE in clinical practice and will therefore need to prescribe metronidazole, it is important that they recognize the condition and its potential complications. With increased awareness, early diagnosis of MRI and discontinuation of metronidazole may become feasible when such patients are referred. Furthermore, we recommend that patients treated with metronidazole should undergo careful and constant surveillance after starting with the medication.

## Abbreviations

DWI: Diffusion weighted imaging
FLAIR: Fluid-attenuated inversion recovery
MIE: Metronidazole-induced encephalopathy
MRI: Magnetic resonance imaging
